# The Levels of Tissue Factor Pathway Inhibitor in Sepsis Patients Receiving Prophylactic Enoxaparin

**DOI:** 10.4274/tjh.2014.0312

**Published:** 2016-05-16

**Authors:** Hadil A. Al Otair, Abdel Galil M. Abdel Gader, Syed M. Khurshid, Abdulaziz H. Alzeer, Abdul Kareem Al Momen, Mashael Al Shaikh, Farja Al Gahtani, Zohair A. Al Aseri, Hossam A.H. Abdelrazik

**Affiliations:** 1 King Saud University College of Medicine, King Khalid University Hospital, Department of Critical Care, Riyadh, Saudi Arabia; 2 King Saud University College of Medicine, King Khalid University Hospital, Department of Physiology, Riyadh, Saudi Arabia; 3 King Saud University College of Medicine, King Khalid University Hospital, Department of Medicine, Riyadh, Saudi Arabia; 4 King Saud University College of Medicine, King Khalid University Hospital, Department of Pharmacy, Riyadh, Saudi Arabia; 5 King Saud University College of Medicine, King Khalid University Hospital, Department of Emergency, Riyadh, Saudi Arabia

**Keywords:** Coagulation, sepsis, Enoxaparin

## Abstract

**Objective:**

Sepsis syndrome is usually accompanied by activation of blood coagulation mechanisms. Earlier studies found deficiencies of the 3 main natural anticoagulants, antithrombin, protein C, and protein S. However, none of these inhibitors block tissue factor, the prime trigger of coagulation during sepsis that is controlled specifically by the tissue factor pathway inhibitor (TFPI). The aim of this study was to characterize the fluctuations in the levels of natural anticoagulants, particularly TFPI, in the course of sepsis and to find out their association with the anticoagulant action of the low-molecular-weight heparin enoxaparin.

**Materials and Methods:**

We studied 51 consecutive patients with sepsis. Blood samples were collected from patients at baseline (0 h) and at 4, 12, and 24 h after enoxaparin administration. The following assays were undertaken using commercial kits: activated partial thromboplastin time, prothrombin time, thrombin time, total and free TFPI, protein C and protein S, antithrombin, fibrinogen, and anti-factor Xa.

**Results:**

Before enoxaparin administration, there was significant prolongation of the prothrombin time and activated partial thromboplastin time, and this remained the case in the 3 subsequent samples. There was marked reduction in the levels of antithrombin, protein C, and total and free protein S to below control values throughout the study. In contrast, plasma levels of both total and free TFPI were markedly elevated and increased after enoxaparin therapy. Anti-factor Xa levels were within the therapeutic range throughout. There was no difference in TFPI levels between those patients who died and those who survived.

**Conclusion:**

Sepsis triggered marked release of TFPI from endothelial cells. This persisted and was increased further following the administration of enoxaparin. In contrast, there was marked consumption of the natural coagulation inhibitors antithrombin, protein C, and protein S. These results go some way towards explaining why the therapeutic use of recombinant TFPI fails to correct sepsis-associated coagulopathy.

## Abstract (tr)

**Amaç:**

Sepsis sendromuna genellikle kan pıhtılaşma sisteminin aktivasyonu eşlik eder. İlk çalışmalar ana doğal 3 antikoagülan olan antitrombin, protein C ve protein S eksikliği bulmuştur. Bununla birlikte, bu inhibitörlerin hiç biri doku faktörü bloke etmez, sepsis sırasındaki koagülasyon tetiklenişi özelllikle doku faktör yolak inhibitörü (DFYİ) ile kontrol edilir. Bu çalışmanın amacı sepsis sırasındaki doğal antikoagülan ve özellikle DFYİ düzeyi dalgalanmalarını karakterize etmek ve bunların düşük moleküler ağırlıklı heaprin enoksaparinin antikoagülan eylemi ile ilişkilerini öğrenmekti.

**Gereç ve Yöntemler:**

Ardışık 51 sepsis hastası çalışmaya alındı. Taban (0 saat) ve enoksaparin verimesinden 4, 12, 24 saat sonra kan örnekleri alındı. Aşağıdaki deneyler ticari kitleri kullanılarak yapılmıştır; parsiyel tromboplastin zamanı, protrombin zamanı, trombin zamanı, toplam ve serbest DFYİ, protein C ve protein S, antitrombin, fibrinojen, ve aktif anti-faktör Xa.

**Bulgular:**

Enoksaparin uygulamadan önce ptorombin zamanı ve aktif parsiyel protrombin zamanında önemli uzama vardı. Bu durum sonraki 3 örneklemde de devam etti. Çalışma boyunca antitrombin, protein C, toplam ve serbest protein S seviyeleri değerlerinde kontrollere göre belirgin bir azalma oldu. Buna karşılık, hem toplam hem de serbest plazma DFYİ değerleri belirgin biçimde yükseldi ve enoksaparin tedavisinden sonra arttı. Anti faktör Xa düzeyleri terapötik aralık içindeydi. Vefat eden ve sağ kalan hastalar arasında DFYİ düzeyi açısından fark yoktu.

**Sonuç:**

Sepsis, endotel hücrelerinden belirgin DFYİ salınımı ile tetiklenir. Bu, enoksaparin uygulmasını takiben kalıcı olmuş ve daha da artmıştır. Bunun aksine, doğal koagülasyon inhibitörleri antitrombin, protein C ve protein S’nin belirgin tüketimi vardı. Bu sonuçlar, tedavi amaçlı rekombinant DFYİ kullanımının sepsis ilişkili koagülopatiyi düzeltmek için neden başarısız olduğunu doğru biçimde açıklamaktadır.

## INTRODUCTION

Sepsis syndrome results from a host reaction to infection that triggers the systemic inflammatory response syndrome, which, on one hand, activates procoagulation mechanisms, and, on the other, shuts down fibrinolysis, leading to the formation of fibrin microthrombi in microcirculation and multiple organ failure [1,2]. In its worst form, the interaction between inflammation and the coagulation system may lead to the development of disseminated intravascular coagulation [3,4].

Over the last 3 decades, numerous reports have emerged that describe disturbances in the measured levels of coagulation parameters in patients with sepsis [5,6,7,8,9]. Much emphasis has been focused on the deficiencies of the 3 main natural coagulation inhibitors: antithrombin (AT), activated protein C, and tissue factor pathway inhibitor (TFPI) [9,10,11,12]. This led to numerous clinical therapeutic trials of administering these inhibitors to patients with sepsis. Some success was initially obtained with the administration of activated protein C, but later on, the PROWESS-SHOCK trial showed an increased risk of bleeding with the use of activated protein C, with no mortality benefit. Similarly, trials with AT and recombinant TFPI generated disappointing results [13,14].

The resultant procoagulant state associated with sepsis has also been recognized as an important risk factor for venous thromboembolism in critically ill patients [14,15]. Therefore, deep vein thrombosis prophylaxis is considered of utmost importance and is practiced with vigilance in intensive care units (ICU) using unfractionated heparin and low-molecular-weight heparin (LMWH) [16,17]. LMWH exerts its antithrombotic effect mainly by inhibiting activated factor X (FXa) and to a lesser degree AT [18]. Nevertheless, failure of deep vein thrombosis prophylaxis in critically ill patients has been well described [17,19]. The reason for this is thought to be multifactorial and one possible proposed explanation could be related to lower anticoagulant effect (as assessed by anti-FXa activity) in these patients, despite appropriate LMWH dosage [20].

The recent availability of more precise assay techniques for the measurement of the natural anticoagulants, particularly total and free TFPI and protein S, encouraged us to monitor the fluctuations of natural anticoagulants in patients with sepsis, in a way that no study has done before, to find a possible explanation for why past trials administering natural anticoagulants to patients with sepsis failed.

Therefore, the aim of this study was to assess the levels of natural anticoagulants, particularly total and free TFPI, in patients with sepsis and septic shock and to find out the association between these fluctuations and the anticoagulant action of the LMWH enoxaparin.

## MATERIALS AND METHODS

### Study Population

Fifty-one consecutive patients were studied; 29 were male and 22 female, with a mean age of 51±20.8 years. All were admitted to the ICU of King Khalid University Hospital, Riyadh, with sepsis or septic shock. Sepsis is defined as systemic inflammatory response syndrome due to infection [1,2]. Septic shock is defined as severe sepsis-induced hypotension that persists despite adequate fluid resuscitation [1,6]. Exclusion criteria were patients younger than 18 years old, body weight of <45 kg or >148 kg, renal insufficiency (creatinine clearance of <30 mL/min), active bleeding, platelet count of <75,000 mm3, INR of >2, activated partial thromboplastin time (APTT) of >2 times the upper normal, therapeutic anticoagulation, pregnancy, porcine hypersensitivity, and administration of unfractionated heparin or LMWH prior to enrollment in the study. Controls (n=42) were healthy individuals (28 males) selected from blood donors, academic staff, and volunteers from the general public. Their ages ranged from 21 to 62 years (mean: 47.4). They were not taking any form of medication at the time of blood sampling.

The study was approved by the Institutional Review Board of the College of Medicine-King Saud University. Written informed consent was obtained from all patients or their next of kin.

### Data Collection

A data entry form was used for the collection of patients’ demographic data and clinical information as well as laboratory results.

Enoxaparin (Clexane R, Aventis Pharma, Frankfurt, Germany), which is a LMWH (4500 Da) isolated from porcine intestinal mucosa and used as sodium salt, was injected subcutaneously at a dose of 0.5 mg/kg in the thighs of all eligible patients after obtaining the baseline blood samples within 1 h of the diagnosis of sepsis [20,21].

Measurements of coagulation tests for APTT, prothrombin time (PT), and thrombin time (TT), as well as the levels of natural anticoagulants including total and free TFPI, protein S, protein C, and AT, were repeated 4, 12, and 24 h after the administration of enoxaparin.

### Blood Collection and Processing

A total of 9.5 mL of blood was carefully collected into vacutainer tubes containing 0.5 mL of sodium citrate (3.8%, 0.129 mol/L; Terumo, Tokyo, Japan) at 0 h, before the administration of the first enoxaparin dose (the baseline sample), and at 4, 12, and 24 h thereafter. Blood samples were mixed gently and transferred immediately to the Coagulation Research Laboratory, Physiology Department, College of Medicine, King Saud University.

The blood sample tubes were centrifuged at 3000 rpm (1000×g) for 15 min in a refrigerated (4-6 °C) centrifuge (Jouan Centrifuge Series, France). Platelet-poor plasma was separated using plastic pipettes and aliquots and immediately stored at -80 °C, until analysis in batches at a later date. Before assays were performed plasma specimens were thawed at 37 °C for 15 min.

### Laboratory Assays

Coagulation screening tests included APTT, PT, and TT. PT was measured using a Stago STA Analyzer and STA Neoplastine CI 5 (freeze-dried rabbit brain thromboplastin with heparin inhibitor). For APTT, the STA PTT Automate 5 Kit was used. TT was measured using the STA thrombin kits with calcium thrombin reagent (approximately 1.5 NIH U/mL, freeze dried). The coefficient of variation (CV) varied from 5% for APTT to 2% for PT and TT. Plasma fibrinogen was measured by a turbidimetric method [22] and the CV varied between 6% and 8%. Anti-FXa was assayed by a colorimetric kit (Rotachrom HBPM/LMWH Assay, Diagnostica Stago, Asnières-sur-Seine, France).

Coagulation inhibitors were assayed using an automated coagulometer (Stago STAT 4) and reagents were supplied by Diagnostica Stago, Asnières-sur-Seine, France: TFPI [Asserachrom Enzyme-Linked Immunosorbent Assay (ELISA) Kit] [23], total and free protein S (Asserachrom Protein S ELISA Kit), protein C (Asserachrom Protein C ELISA Kit), and AT (Stachrom Antithrombin Kit), with CV of 5% or less for total TFPI, free TFPI, total protein S, free protein S, and protein C and 4% for AT. STA-Liquid Anti-Xa for use with the STA Compact (Diagnostica Stago, France) was used for the quantitative determination of the potentiating effect of LMWH on antithrombin by recording the anti-FXa activity in plasma using a chromogenic substrate. Results were expressed as percent activity and according to the manufacturer’s instructions.

### Statistical Methods

The Mann-Whitney U test was used to compare means for 2 independent groups. The chi-square test or Fisher’s test was used as appropriate to compare the percentages for 2 categorical variables. A p-value of less than 0.05 indicated statistical significance. SPSS 15 for Windows was used for the analysis and for the drawing of the bar graphs.

## RESULTS

Pneumonia was the most common diagnosis (37.2%) in the study population, followed by urosepsis and abdominal sepsis (11.8% each). Thirteen patients (25.5%) developed septic shock and were started on vasopressors. Nine (69.2%) of them died during hospitalization, 10 died during the first week, and 6 patients died 2 weeks later. None died during the study period. This accounted for a mortality rate of 31.4% (Table 1). A definite infective organism was identified in 22 patients.

To facilitate comparisons between subjects and to reduce the day-to-day variation in individuals, the results of each test were expressed as percentage of normal pooled plasma.

**Figure 1:**
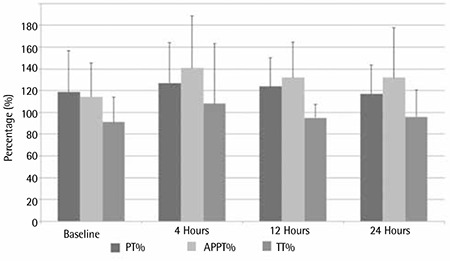
Comparison of PT%, APTT%, and TT% at baseline and at 4, 12, and 24 h after the administration of enoxaparin.

On arrival to the Accident and Emergency Department and before receiving any treatment, the baseline blood tests showed prolongation of PT and APTT; the TT did not fluctuate significantly. Significant prolongation of both PT and APTT persisted in the 3 subsequent samples (4, 8, and 24 h), with the APTT prolongation getting worse in the subsequent samples (Figure 1).

**Figure 2:**
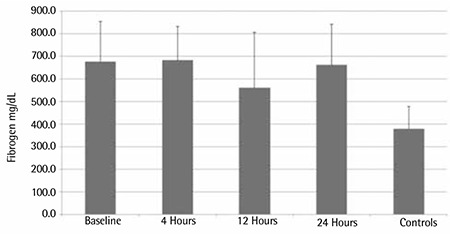
Comparison of fibrinogen at baseline and at 4, 12, and 24 h after the administration of enoxaparin.

The plasma fibrinogen levels were significantly elevated above normal control values at baseline and in the 3 subsequent samples (local laboratory reference values: 150-400 mg/dL) (Figure 2).

**Figure 3:**
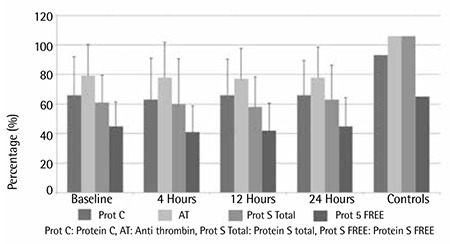
Comparison of plasma levels of protein C, AT, and total and free protein S at baseline and at 4, 12, and 24 h after the administration of enoxaparin.

There was significant reduction in the levels of the natural anticoagulants AT, protein C, and total and free protein S below control values from baseline and in the 3 subsequent samples (4, 12, and 24 h) (Figure 3).

**Figure 4:**
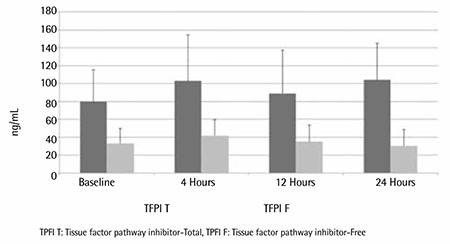
Comparison of the plasma levels of total and free TFPI at baseline and at 4, 12, and 24 h after the administration of enoxaparin.

In contrast to the above 3 natural coagulation inhibitors, the plasma levels of total and free TFPI were markedly elevated above control values (local laboratory reference value: 60.7±16.9 ng/mL) throughout the study period. The mean level of total TFPI was 73.0±39.0 ng/mL at baseline, and it remained significantly elevated at 4 h (101.9±55.5 ng/mL), 12 h (91.2±55.1 ng/mL), and 24 h (85.7±55.5 ng/mL). A similar trend was noted in the fluctuations of free TFPI, whose levels were also elevated significantly, but much more so than total TFPI, to almost 4 times the control levels upon arrival to the Accident and Emergency Department (30.0±17.1 ng/mL) (Figure 4).

When the patients who had sepsis (n=38) were compared to patients with septic shock (n=13), we noted higher PT after 4 h and higher free TFPI after 4 and 12 h of enoxaparin administration (Table 2).

The plasma level of anti-FXa at 4 h was 0.52±0.11 IU/mL, at 12 h was 0.5±0.07 IU/mL, and at 24 h was 0.59±0.11 IU/mL; all were within the prophylactic range (0.2-0.5 IU/mL) [24].

Comparing the measured hemostatic variables in survivors and nonsurvivors, there were only 3 isolated significant findings: lower levels in nonsurvivors of TT (15.9±2.48% in nonsurvivors versus 21.74±10.2% in survivors, p=0.04), AT (74.36±17.6% in nonsurvivors versus 106.5±22.59%, p=0.01), and protein C (62.51±21.19% in nonsurvivors versus 90.0±26.87%, p=0.03) in the 24-h samples.

## DISCUSSION

The findings of the current study revealed marked derangement of the coagulation system in patients with sepsis and septic shock, in the form of significant prolongation of results of both the screening tests of the intrinsic (APTT) and extrinsic (PT) coagulation pathways that persisted after enoxaparin administration. There was also very significant consumption of the natural anticoagulants protein C, AT, and total and free protein S. On the other hand, we noted with much interest that the baseline levels of both total and free TFPI were elevated above healthy control values and increased further after the administration of enoxaparin.

Numerous previous studies have examined the fluctuations of the circulating levels of hemostatic parameters in septic syndrome. In this respect, natural coagulation inhibitors AT, activated protein C, TFPI, and thrombomodulin received much attention and almost all studies found marked reduction in their blood levels [3,4]. Our study is in accordance with these studies and showed lower levels of AT, protein C, and total and free protein S at baseline and 4, 12, and 24 h after enoxaparin administration.

AT is the main inactivator of thrombin and also inhibits the activated forms of FIX, FX, and XI. Protein C, in the presence of protein S, inhibits the activated forms of FVIII and FV. In our patients, the levels of these inhibitors remained below control values throughout the study period, which suggested their consumption in the face of the activated coagulation in these septic patients. However, none of these 3 inhibitors act on tissue factor, the prime trigger of coagulation in vivo [25] and whose expression in septic patients is markedly enhanced by proinflammatory cytokines on the surface of endothelial cells and monocytes [9,11,25,26].

The prime and specific physiological inhibitor of tissue factor is TFPI, which is a proteinase inhibitor generated mainly from the microvascular endothelium and that circulates in 2 forms: 80% bound to lipoproteins and 20% in the physiologically active free form [26,27]. TFPI also inhibits FXa directly and indirectly by blocking action of the FVIIa/TF complex [10,27].

In an early report, Gando et al. [25], who measured both TF and TFPI daily for 4 days, concluded that tissue factor production is not balanced by concurrent production of TFPI and that underlies the resulting activation of the coagulation system. The design of the current study is different from that of Gando et al. [25] and we have undertaken multiple measurements over the first 24 h of admission, which we think is a critical period in the management of septic patients. We also carried out more detailed measurements of both total TFPI and free TPFI [26].

We noted with much interest the remarkable elevation in the levels of both total and free TFPI above the healthy control levels on admission, indicating that the prime inhibitor of the tissue factor does in fact show a very active response to the presumed excessive sepsis-induced generation of tissue factor.

The administration of prophylactic doses of enoxaparin resulted in significant inhibition of FXa at 4, 12, and 24 h and was associated with further rise in the circulating levels of both forms of TFPI. Free TFPI exhibited more remarkable elevation (4 times the control levels) than total TFPI. This was taken to indicate that sepsis, in its own right, must be a strong trigger to the release of TFPI from the vascular endothelium. This release process must have approached its maximum degree following the administration of enoxaparin, which is known to be most potent in the mobilization and release of TFPI from the vascular endothelium as compared to other LMWHs [28,29]. Interestingly, some studies have reported not only reduced levels of natural coagulation inhibitors but also impairment of their function [2,28]. If this is indeed the case, and until the mechanism of this impairment of function is delineated, no benefit should be expected of the therapeutic uses of genetically engineered recombinant natural coagulation inhibitors.

In the present study, we noted a trend towards higher TFPI levels and particularly free TFPI in patients with septic shock as compared to patients with sepsis. This could represent a more exaggerated release of TFPI in these patients with more severe disease. However, the number of patients in this group was small and perhaps future studies with larger number of patients with septic shock are needed to confirm these observations.

Unlike other studies, in which most of the patients were receiving vasopressors [30,31], we found that enoxaparin administered in prophylactic doses resulted in significant inhibition of FXa. This suggests the presence of additional factors that contribute to the failure of deep venous thrombosis prophylaxis in patients with sepsis. One possibility could be the lower levels of Na as described before [9,10,11,12] and confirmed by our findings.

In conclusion, the main finding of the current study is the remarkable elevation in the plasma levels of both total and free TFPI in septic patients at baseline. The levels of both forms of the inhibitor remained elevated throughout the first 24 h with further elevation after enoxaparin administration. This observation would help to explain why the administration of recombinant TFPI did not affect the course and outcome of sepsis and septic shock.

## Ethics

Ethics Committee Approval: The study was approved by the Institutional Review Board of the College of Medicine-King Saud University. Informed Consent: A written informed consent was obtained from all patients or their next of kin.

## Figures and Tables

**Table 1:**
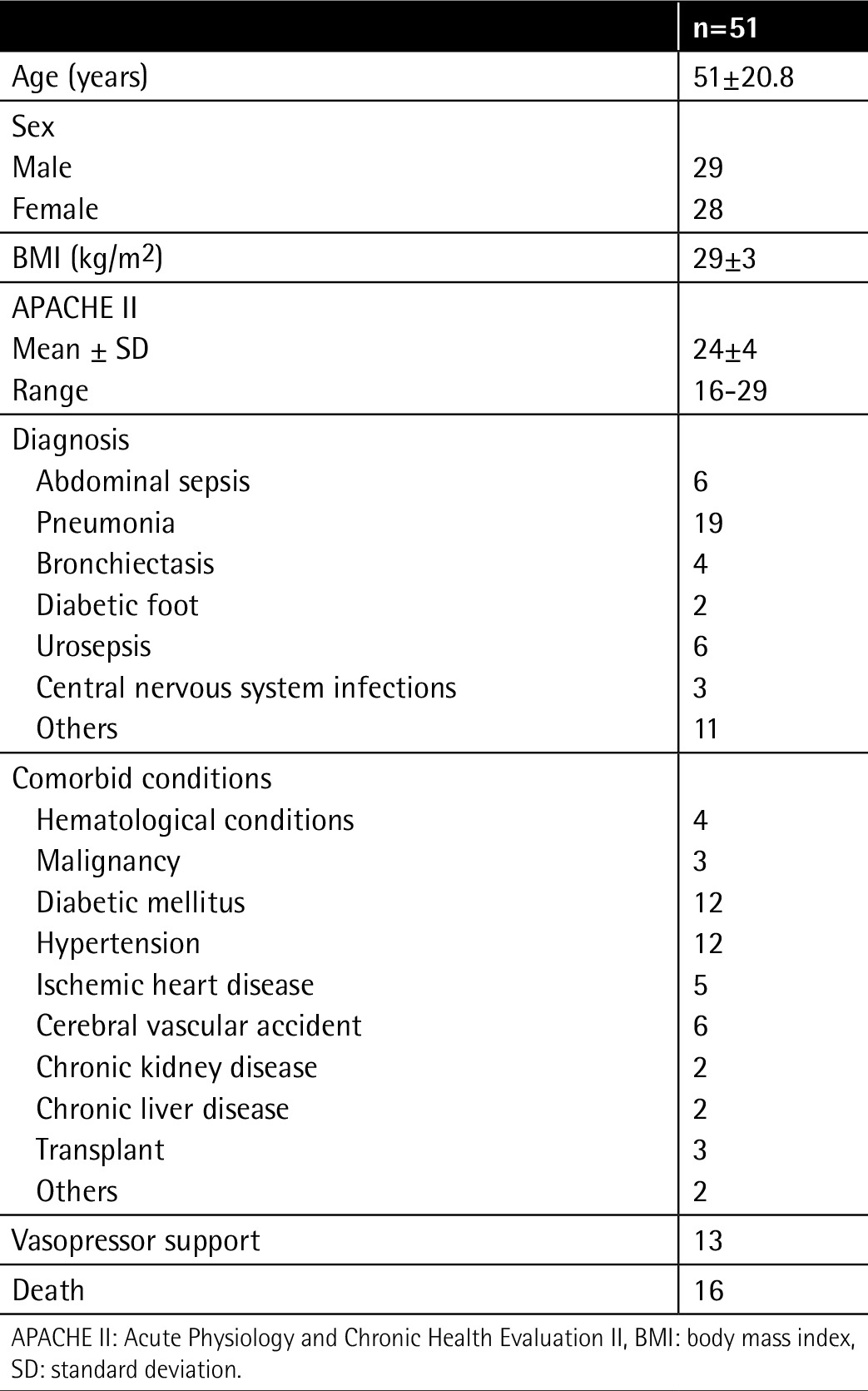
Descriptive statistics of study population.

**Table 2:**
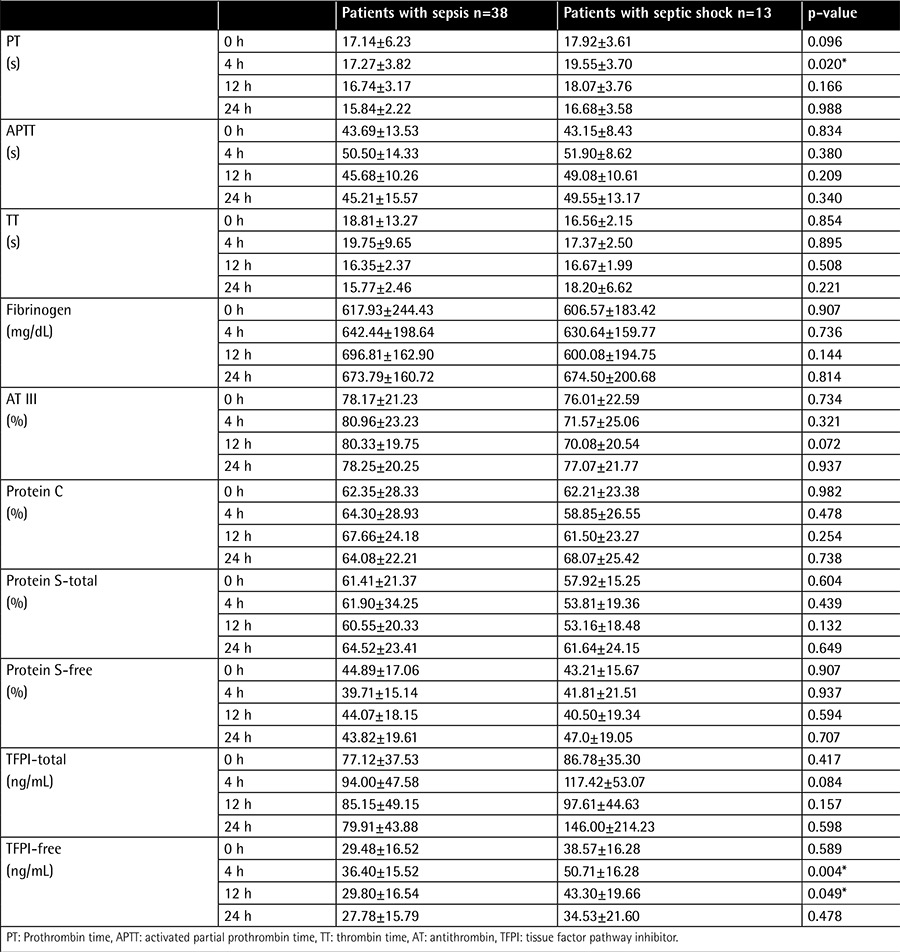
Comparison between the hemostatic variables in patients with sepsis and septic shock.
